# Synthesis and Fluorescence Properties of Structurally Characterized Heterobimetalic Cu(II)–Na(I) Bis(salamo)-Based Complex Bearing Square Planar, Square Pyramid and Triangular Prism Geometries of Metal Centers

**DOI:** 10.3390/molecules23051006

**Published:** 2018-04-25

**Authors:** Xiu-Yan Dong, Qing Zhao, Zhi-Li Wei, Hao-Ran Mu, Han Zhang, Wen-Kui Dong

**Affiliations:** School of Chemical and Biological Engineering, Lanzhou Jiaotong University, Lanzhou, Gansu 730070, China; dxy568@163.com (X.-Y.D.); zq18215194507@163.com (Q.Z.); wzl254452590@163.com (Z.-L.W.); muhaoran121@163.com (H.-R.M.); 13572510846@163.com (H.Z.)

**Keywords:** symmetric bis(salamo)-type tetraoxime, heterobimetalic complex, synthesis, crystal structure, spectroscopic study

## Abstract

A novel heterotrinuclear complex [Cu_2_(L)Na(*µ*-NO_3_)]∙CH_3_OH∙CHCl_3_ derived from a symmetric bis(salamo)-type tetraoxime H_4_L having a naphthalenediol unit, was prepared and structurally characterized via means of elemental analyses, UV-Vis, FT-IR, fluorescent spectra and single-crystal X-ray diffraction. The heterobimetallic Cu(II)–Na(I) complex was acquired via the reaction of H_4_L with 2 equivalents of Cu(NO_3_)_2_·2H_2_O and 1 equivalent of NaOAc. Clearly, the heterotrinuclear Cu(II)–Na(I) complex has a 1:2:1 ligand-to-metal (Cu(II) and Na(I)) ratio. X-ray diffraction results exhibited the different geometric behaviors of the Na(I) and Cu(II) atoms in the heterotrinuclear complex; the both Cu(II) atoms are sited in the N_2_O_2_ coordination environments of fully deprotonated (L)^4−^ unit. One Cu(II) atom (Cu1) is five-coordinated and possesses a geometry of slightly distorted square pyramid, while another Cu(II) atom (Cu2) is four-coordination possessing a square planar coordination geometry. Moreover, the Na(I) atom is in the O_6_ cavity and adopts seven-coordination with a geometry of slightly distorted single triangular prism. In addition, there are abundant supramolecular interactions in the Cu(II)–Na(I) complex. The fluorescence spectra showed the Cu(II)–Na(I) complex possesses a significant fluorescent quenching and exhibited a hypsochromic-shift compared with the ligand H_4_L.

## 1. Introduction

A wide variety of N_2_O_2_–donor chelate ligands have been proverbially used to prepare alkaline earth, rare earth, and transition metal complexes for several decades. More recently, modified salen-type ligands having imine groups (–C=N–) [1,2,3,4,5,6,7,8], bear the potentially tetradentate N_2_O_2_-donor and can form relatively stable complexes with different metal ions. Salen and its derivatives are extremely attractive and commonly versatile ligands because their metal complexes have been studied in potential application in catalyses [9,10], supramolecular architectures [11,12,13,14,15,16,17,18,19], luminescence properties [20,21,22,23,24,25,26], electrochemical conducts [27,28], biological systems [29,30,31,32,33,34,35,36,37], optical sensor [38,39], magnetic materials [40,41,42,43,44,45] and nonlinear optical materials [46], and so forth. A lot of researches have been fulfilled to design and synthesize mono-nuclear and homo- or hetero-polynuclear metal complexes possessing salen-type or salamo-type ligand or its analogues. Compared to salen, salamo-type ligand [47,48,49,50,51,52,53,54,55,56] has a –CH=N–O–(CH)_n_–O–N=CH– configuration and its corresponding complexes are more stable relatively.

**Figure 1:**
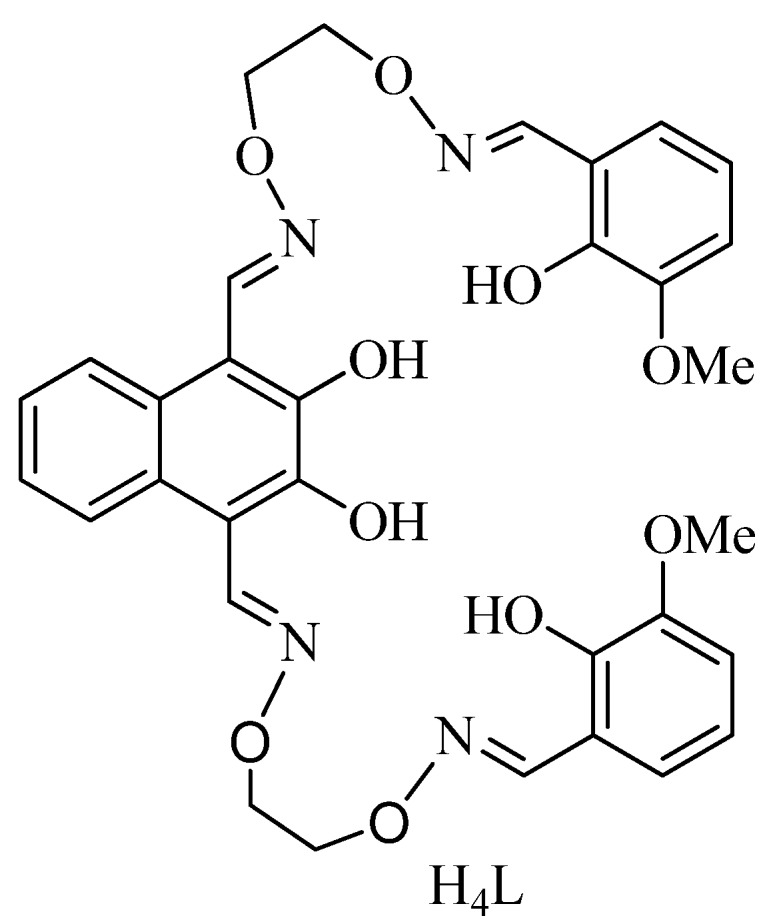
Structural representation of H_4_L.

The bis(salamo)-type ligand H_4_L containing O_6_ coordination sphere was already prepared earlier to obtain a series of Zn(II)–M(II), Co(II)–M(II) (M = Ca, Sr and Ba) and Zn(II)–RE(III) (RE = La, Ce and Dy) complexes. Therefore, these compounds are considered to be heterotrinuclear bis(salamo)-type complexes. Meanwhile, the crystal structures and properties of the obtained complexes have been studied in detail [28,57,58,59]. Owing to the bis(salamo)-type ligand H_4_L bearing an O_6_ coordination sphere, it can form homotrinuclear bis(salamo)-type complexes. The transition metal atom located in O_6_ coordination sphere can be replaced by alkali metal atoms with a larger atomic radius. To study the structural characteristics and fluorescent behaviors of heterobimetalic complexes of bis(salamo)-type ligand, herein we have designed and prepared a novel heterobimetalic complex [Cu_2_(L)Na(*µ*-NO_3_)]∙CH_3_OH∙CHCl_3_ derived from the bis(salamo)-type tetraoxime H_4_L containing a naphthalenediol unit. The structural representation of H_4_L is shown in Figure 1. Most importantly, the spectroscopic studies of the Cu(II)–Na(I) complex were investigated.

## 2. Results and Discussion

### 2.1. IR Spectra

The main FT-IR absorption bands characteristic for H_4_L and its Cu(II)–Na(I) complex from 500 to 4000 cm^−1^ regions are shown in Table 1.

Generally, the typical O–H stretching band is usually observed at the 3500–3100 cm^−1^ region. A broad band of medium intensity was found around 3172 cm^−1^ in the free ligand H_4_L, this is an evidence for the stretching vibration of the O–H groups. However, this band disappeared in the Cu(II)–Na(I) complex. H_4_L showed a typical C=N stretching band occurring at 1634 cm^−1^, while the Cu(II)–Na(I) complex showed this band at 1618 cm^−1^. In contrast to the free ligand H_4_L it is moved to a lower wavenumber by 16 cm^−1^ due to coordination of the N atoms of the C=N groups to the Cu(II) atoms [57]. This fact is also approved by the appearance of new bands within the wavenumber at 565 cm^−1^ due to *v*(Cu(II)-N) vibrations. The characteristic absorption band of the Ar–O group occurred at 1241 cm^−1^ in H_4_L. However, the Ar–O stretching band in the Cu(II)–Na(I) complex was moved to a lower wavenumber at ca. 9 cm^−1^, exhibiting that the M–O bonds are formed between the metal atoms and the methoxy and phenolic O atoms of H_4_L [60]. The facts mentioned above fare in agreement with the results of X ray diffraction.

### 2.2. UV-Vis Spectra

In this study, UV-Vis spectra of H_4_L in CHCl_3_:CH_3_OH (1:1) (*c* = 2.5 × 10^−5^ mol L^−1^) with its Cu(II)–Na(I) complex in CH_3_OH:H_2_O (10:1) (*c* = 1 × 10^−3^ mol L^−1^) in freshly prepared solution are obtained in the range of 250–600 nm.

It can be seen from the absorption spectrum of H_4_L, there are four continuous absorption peaks at about 269, 342, 360 and 378 nm [57]. The peak at 269 nm can be attributed to the π-π* transition of the benzene rings while the other three peaks can be assigned to the π-π* transition of the oxime groups [57,61].

**Figure 2:**
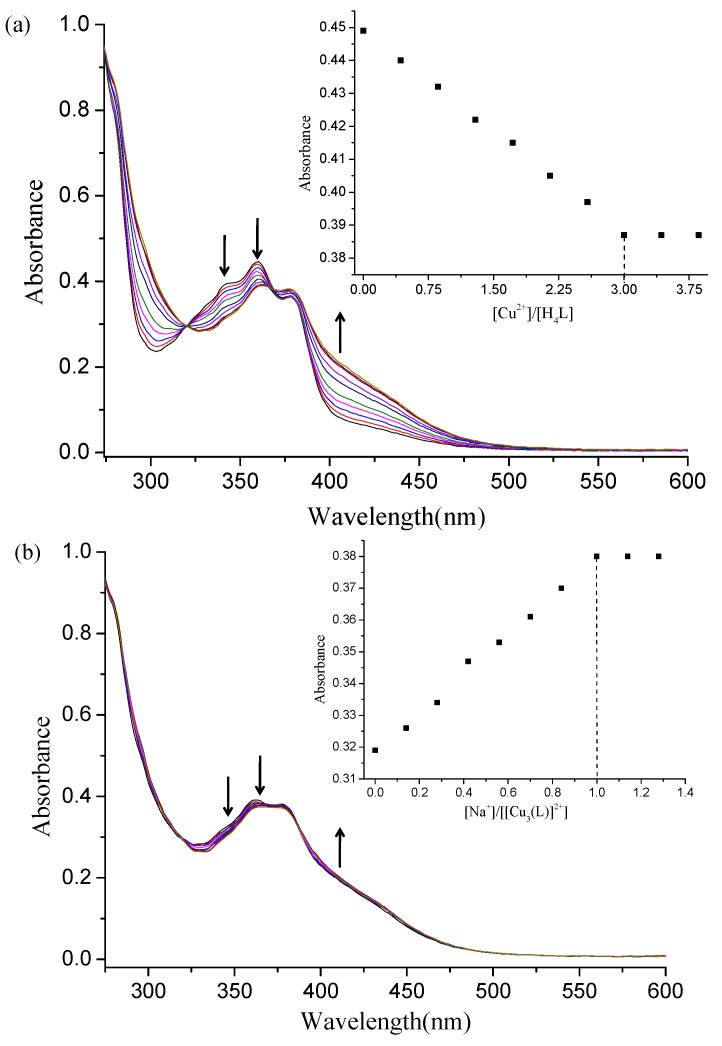
(**a**) UV-Vis spectral changes of H_4_L (2.5 × 10^−5^ M) on addition of Cu(II) ions (1.0 × 10^−3^ M); (**b**) UV-Vis spectral changes of the [LCu_3_]^2+^ on addition of Na(I) ions (1.0 × 10^−3^ M).

In the titration experiment of the Cu(II)–Na(I) complex, the gradual addition of Cu(NO_3_)_2_ solution aroused the changes of absorption peaks. Compared to H_4_L, the peaks are bathochromically shifted [62]. This fact mentioned above is owing to the coordination of the Cu(II) ion with H_4_L. When 3 equiv of the Cu(II) ions were added to the solution of H_4_L, the absorbance of the solution no longer changed. The spectral titration obviously revealed the formation of a 1:3 (ligand-to-Cu(II) ratio) Cu(II) complex (Figure 2a).

What is more, the gradual additions of NaOAc solution were continuously added the above solution. Upon addition of 1 equiv of Na(I) ions, the solution absorbance basically remains stable. Clearly, the spectral titration obviously indicated that the ratio of the replacement reaction stoichiometry is 1:1 and is depicted in Figure 2b.

### 2.3. Crystal Structure Description

**Figure 3:**
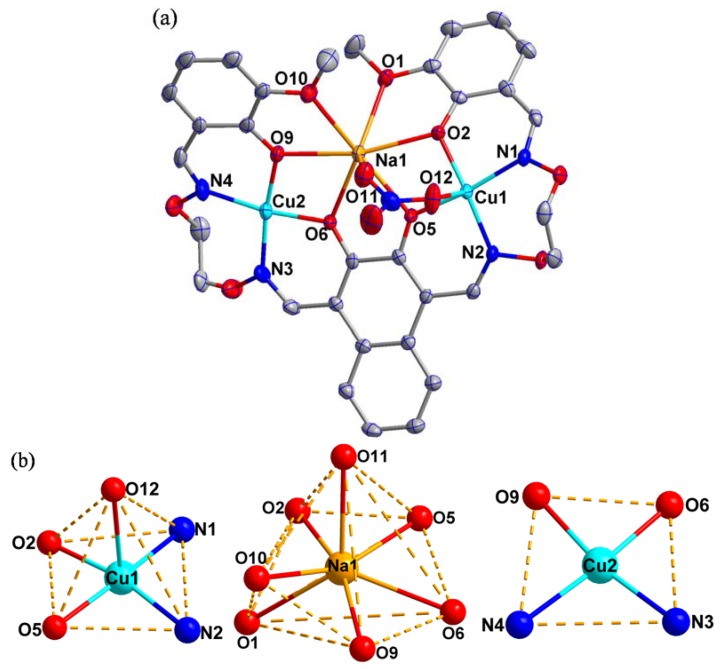
(**a**) Molecular structure of the Cu(II)–Na(I) complex (H atoms, crystallizing methanol and chloroform molecules are omitted for clarity, and thermal ellipsoids are drawn at the 30% probability level); (**b**) Coordination polyhedrons for Cu(II) and Na(I) atoms.

The structure of the Cu(II)–Na(I) complex was characterized by X-ray crystallography, and is depicted in Figure 3.

It is shown that the Cu(II)–Na(I) complex crystallizes in the monoclinic system with space group *P* 2_1_/*n* (Attempting to set the space group to *P*2_1_/*c*, the structure of the complex was not determined, which is determined by the different orientations chosen when determining the crystal cell. The structure of expected complex is obtained when the space group is set to *P*2_1_/*n*, and the bond lengths and angles are all within the expected ranges.) [63] and contains two Cu(II) atoms, one Na(I) atom, one completely deprotonated (L)^4−^ moiety, one *µ*-NO_3_ ion, one crystallizing methanol and chloroform molecules, possessing a hetero-trinuclear structure. According to our design, the bis(salamo)-type ligand H_4_L has an O_6_ coordination sphere which is usually occupied by a larger radius metal ion [64]. Therefore, we consider the complex obtained to be a heterotrinuclear Cu(II)–Na(I) complex and not sodium salt of the Cu(II) complex [65]. At the same time, it is shown that the ligand-to-metal (Cu(II) and Na(I)) ratio is 1:2:1. This structure in this paper is different from the structure reported earlier, in which the two Cu(II) atoms are located in N_2_O_2_ sites, and one Cu(II) atom is still bonded to a methanol molecule, while the other Cu(II) atom is bonded to one O atom of a *μ*-NO_3_ ion. Finally, both Cu(II) atoms adopt five-coordinated and possess geometries of slightly distorted square pyramid. The capped site is occupied by Br3 with the bond length (Na1–Br3, 2.765(3) Å) and the Na-O bond length is in the ranges of 2.324(2)–3.025(3) Å [17].

As shown in Figure 3, the N_2_O_2_ compartments of the fully deprotonated (L)^4−^ moiety are occupied by two Cu(II) atoms (Cu1 and Cu2), while the central Na(I) atom is located at O_6_ cavity. The Cu1 atom is bonded to two N atoms (Cu1–N1, 2.002(3) and Cu1-N2, 1.950(3)) and two O atoms (Cu1–O2, 1.907(2) Å and Cu1-O5, 1.903(2) Å) of the ligand (L)^4−^ moiety forming the square base. Meantime, the Cu1 atom is also coordinated with one O atom (Cu1–O12, 2.462(3) Å) of a bisdentate *μ*-NO_3_ ion. The Cu1 atom is five-coordinated with geometry of a slightly distorted square pyramid, which was deduced by calculating the value of *τ* = 0.0058 [66]. Interestingly, the Cu2 atom is coordinated to the N atoms (Cu2–N3, 1.944(3) Å and Cu2–N4, 1.981(3) Å) and O atoms (Cu2–O6, 1.909(2) Å and Cu2-O9, 1.907(3) Å) of the N_2_O_2_ site. Therefore, the Cu2 atom is four-coordinated and possesses a geometry of slightly distorted square planar. The Na(I) atom occupies central O_6_ site (Na1–O1, 2.546(3) Å; Na1-O2, 2.388(3) Å; Na1-O5, 2.355(3) Å; Na1-O6, 2.454(3) Å; Na1-O9, 2.429(3) Å and Na1-O10, 2.475(3) Å) from four phenolic O and two methoxy O atoms of the ligand (L)^4−^ moiety [17]. Meanwhile, the Na(I) atom is still coordinated to one O atom (Na1-O11, 2.490(4)) of *μ*-NO_3_ ion. In a word, the Na-O bond length is in the range of 2.355(3)–2.546(3) Å, which is in the regular range compared with previously reported data [17]. Therefore, according to our previous report, the Na–O bonds mentioned above could be regarded as coordination bonds. Finally, the Na(I) atom adopts seven-coordinated and possesses a geometry of slightly distorted single triangular prism. The crystallographic data and structure refinement parameters are summed in Table 2. Selected bond lengths and angles are summed in Table 3.

### 2.4. Supramolecular Interactions

Single crystal determination showed that the Cu(II)–Na(I) complex forms a self-assembling infinite supra-molecular structure by C-H⋯π and hydrogen bond interactions. The hydrogen bonds and C-H⋯π stacking interactions are listed in Table 4.

**Figure 4:**
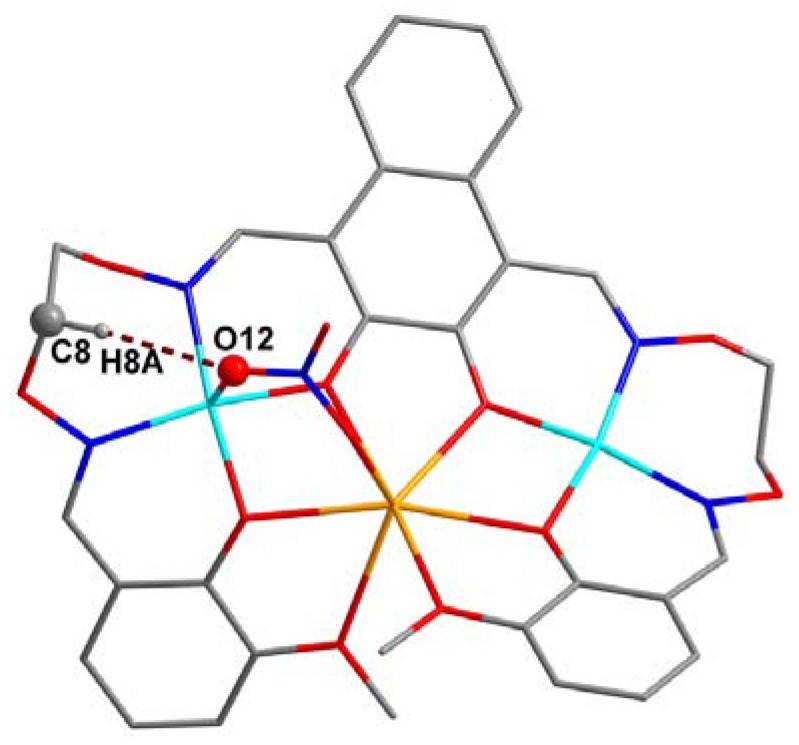
Intra-molecular hydrogen bond interactions of the Cu(II)–Na(I) complex.

**Figure 5:**
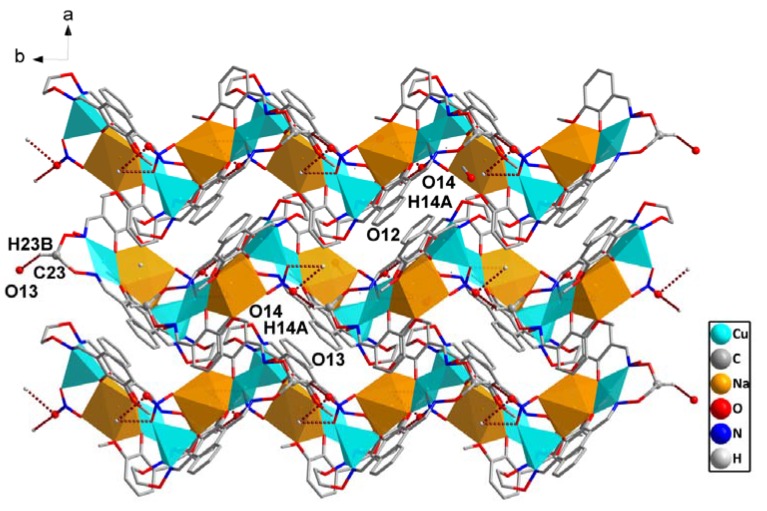
2D supra-molecular structure by inter-molecular O-H⋯O and C-H⋯O interactions in the Cu(II)–Na(I) complex.

**Figure 6:**
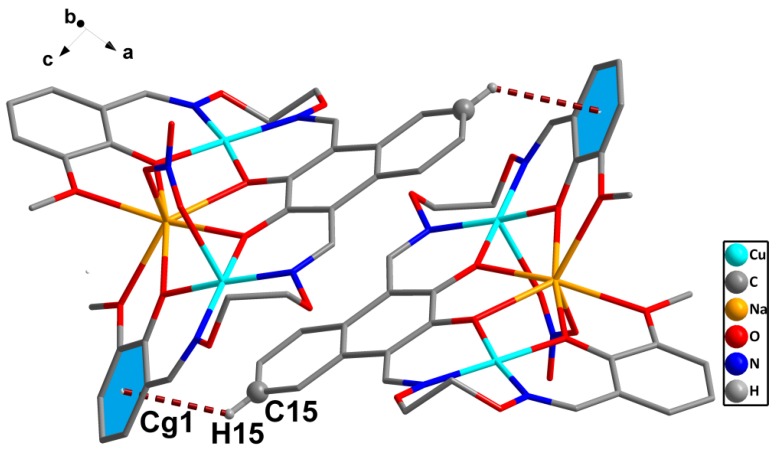
Intermolecular C-H⋯π interactions of the Cu(II)–Na(I) complex.

In the Cu(II)–Na(I) complex, there is one intra-molecular C8-H8A⋯O12 hydrogen bond, showing in Figure 4. As illustrated in Figure 5, an infinite 2D supra-molecular structure is interlinked via three pairs of inter-molecular O14-H14A⋯O12, O14-H14A⋯O13 and C23-H23B⋯O13 hydrogen bonds [67,68,69,70,71,72,73,74,75]. Especially, one significant C15-H15⋯Cg1 (C1-C6) (C-H⋯π) interaction is constructed and shown in Figure 6.

### 2.5. Fluorescence Spectra

The emission spectra of H_4_L in CHCl_3_:CH_3_OH (1:1) (*c* = 2.5 × 10^−5^ mol L^−1^) solution and its Cu(II)–Na(I) complex in CH_3_OH (*c* = 1.0 × 10^−5^ mol L^−1^) were determined at room temperature.

**Figure 7:**
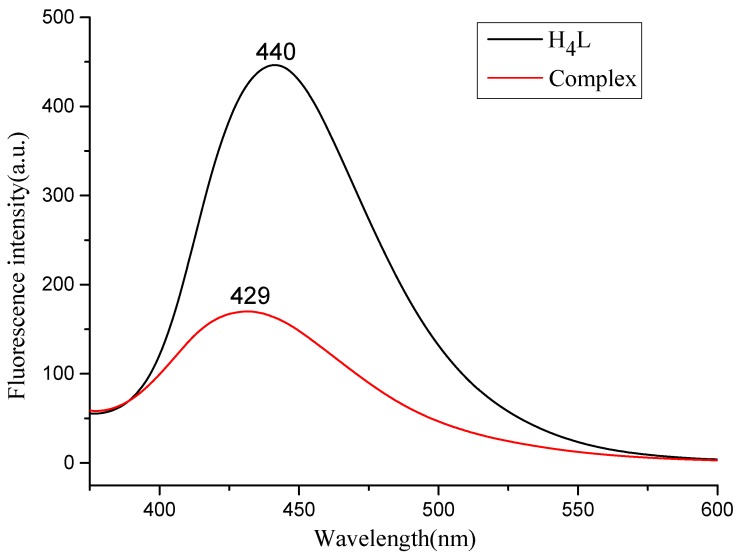
Emission spectra of H_4_L and its Cu(II)–Na(I) complex.

As shown in Figure 7, the ligand H_4_L exhibited one relatively strong emission at 440 nm upon excitation at 330 nm, which should be attributed to the intra-ligand π–π* transition [67]. In comparison with the H_4_L, fluorescent property study of the Cu(II)–Na(I) complex is shown that the fluorescent intensity weakens markedly, exhibiting a broad peak with maximum emission at 429 nm upon excitation at 330 nm. That hypsochromic-shift phenomenon can explain the complexation of H_4_L and metal ions (Cu(II) and Na(I)), which is attributed to ligand-to-metal charge transfer (LMCT) [68].

## 3. Experimental Section

### 3.1. Materials and Methods

2-Hydroxy-3-methoxybenzaldehyde (99%), pyridinium chlorochromate (98%), methyl trioctyl ammonium chloride (90%) and borontribromide (99.9%) were bought from Alfa Aesar (New York, NY, USA). Hydrobromic acid 33 wt % solution in acetic acid was purchased from J&K Scientific Ltd. (Beijing, China). All chemicals and solvents used for the synthesis were of the best available analytical reagent grade, without further purification in the preparation of the free ligand and its complex. Elemental analyses for C, H, and N were carried out using a GmbH Vario EL V3.00 automatic elemental analysis instrument (Elementar, Berlin, Germany). Elemental analyses for Cu and Na atoms were detected with an IRIS ER/S-WP–1 ICP atomic emission spectrometer (IRIS, Elementar, Berlin, Germany). IR spectra were measured on a VERTEX70 FT-IR spectrophotometer (Bruker, Billerica, MA, USA), with samples prepared as KBr (500–4000 cm^−1^) pellets. Melting points were recorded on use of a microscopic melting point apparatus made in Beijing Taike Instrument Limited Company and were uncorrected (Beijing, China). UV-Vis absorption spectra were recorded on a Shimadzu UV-3900spectrometer (Hitachi, Shimadzu, Tokyo, Japan). The fluorescence spectra were taken on Hitachi F-7000 spectrometer (Hitachi, Tokyo, Japan). X-ray single crystal structure determinations were carried out on a Super Nova Dual (Cu at zero) Eos four-circle diffractometer (Bruker, Billerica, MA, USA).

### 3.2. Synthesis of H_4_L

**Scheme 1:**
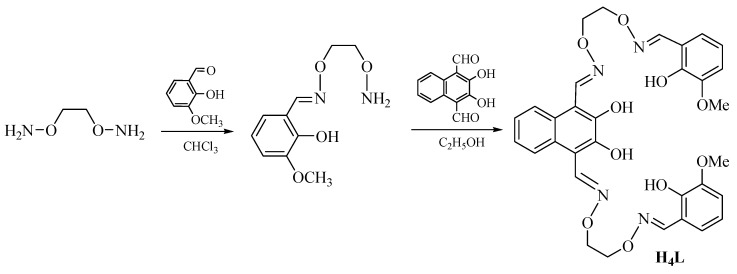
Synthetic route to H_4_L.

The main reaction steps involved in the synthesis of H_4_L was gained via reacting 2,3-dihydroxynaphthalene-1,4-dicarbaldehyde with 3-methoxysalicylaldehyde. 2,3-Dihydroxynaphthalene-1,4-dicarbaldehyde and 2-[*O*-(1-ethyloxyamide)]oxime-6-methoxyphenol were prepared according to an analogous method [28,57,58,59]. The synthesis of H_4_L is given in Scheme 1.

H_4_L was prepared according to the previously reported method [28,57,58,59]. Yield: 60.2%. m.p. 170–171 °C. Anal. Calcd. (%) for C_32_H_32_N_4_O_10_ (632.42): C, 60.75; H, 5.10; N, 8.86. Found (%): C, 60.93; H, 5.23; N, 8.74. ^1^H-NMR (400 MHz, CDCl_3_) *δ*11.03 (s, 2H), 9.82 (s, 2H), 9.14 (s, 2H), 8.29 (s, 2H), 7.97 (q, *J* = 3.2 Hz, 2H), 7.41 (q, *J* = 6.0, 2.9 Hz, 2H), 7.06–6.68 (m, 6H), 4.58 (t, 8H), 3.89 (s, 6H). UV-Vis [in methanol/chloroform (1:1)], λ_max_ (nm) [2.5 × 10^−^5 M]: 269, 342, 360, 378.

### 3.3. Synthesis of the Cu(II)–Na(I) Complex

A mixture solution of Cu(NO_3_)_2_·2H_2_O (4.82 mg, 0.02 mmol) in methanol (2 mL) and NaOAc (0.82 mg, 0.01 mmol) in methanol (2 mL) was added to a stirring solution of H_4_L (6.32 mg, 0.01 mmol) in chloroform (2 mL). The color of the mixing solution immediately turned dark green. The mixture was filtered, and the filtrate was allowed to stay for slow evaporation. After about three weeks, clear light green single-crystals suitable for X-ray diffraction studies were gained. Yield: 40.3%. Anal. Calcd. (%) for C_34_H_33_Cl_3_Cu_2_NaN_5_O_14_ (992.07): C, 41.16; H, 3.35; N, 7.06; Cu, 12.81; Na, 2.32. Found (%): C, 41.33; H, 3.49; N, 6.88; Cu, 12.64; Na, 2.17. UV-Vis [in methanol/H_2_O (10:1)], λ_max_ (nm) [1.0 × 10^−^3 M]: 350, 369, 382.

### 3.4. Crystal Structure Determination and Refinement

Single crystal of dimensions 0.15 × 0.21 × 0.24 mm for the heterotrinuclear Cu(II)–Na(I) complex was mounted on a glass rod. The crystal data were collected with a Super Nova (Dual, Cu at zero, Eos) diffractometer with graphite monochromated Mo *Kα* radiation (λ = 0.71073 Å) at 154.89(10) K. Multiscan absorption corrections were applied. The structure was solved by direct methods and refined anisotropically using full-matrix least-squares methods on *F*^2^ with the SHELX-2014 program package. Nonhydrogen atoms of the compound were refined with anisotropic temperature parameters. The positions of H atoms were calculated and isotropically fixed in the final refinement.

Supplementary crystallographic data have been deposited at Cambridge Crystallographic Data Centre (CCDC No. 1815945) and can be gained free of charge via www.ccdc.cam.ac.uk/conts/retrieving.html.

## 4. Conclusions

Overall, we have designed and synthesized a novel heterobimetallic Cu(II)–Na(I) complex derived from a symmetric bis(salamo)-type ligand H_4_L. X-ray crystallographic investigation of the heterotrinuclear Cu(II)–Na(I) complex revealed there is a 1:2:1 ligand-to-metal (Cu(II) and Na(I)) ratio. The Cu1 atom is five-coordinated and possesses the geometry of slightly distorted square pyramid and the Cu2 atom is four-coordinated and possesses the geometry of square planar. The Na(I) atom adopts seven-coordinated with a slightly distorted single triangular prism geometry. The UV-Vis titration experiment clearly displayed the coordination ratio between the ligand H_4_L and the Cu(II) and Na(I) ions. The fluorescence spectra showed the complex possesses a significant fluorescent quenching, and exhibited a hypsochromic-shift compared with the ligand H_4_L.

## Figures and Tables

**Table 1:** Main FT-IR absorption bands for H_4_L and its Cu(II)–Na(I) complex (cm^−1^).

Compound	*ν*(O–H)	*ν*(C=N)	*ν*(Ar–O)	*ν*(M–N)
H_4_L	3172	1634	1241	–
Complex	–	1618	1232	565

**Table 2:** Crystal and structure refinement data for the Cu(II)–Na(I) complex.

Empirical Formula	C_34_H_33_Cl_3_Cu_2_NaN_5_O_14_
Formula weight	992.07
*T* (K)	154.89(10)
Radiation (Å)	Mo *Kα*, 0.71073
Crystal system	Monoclinic
Space group	*P 2*_1_ */n*
*a* (Å)	15.8326(3)
*b* (Å)	11.4630(2)
*c* (Å)	21.7834(4)
*α* (°)	90
*β* (°)	98.135(2)
*γ* (°)	90
Volume (Å^3^)	3913.68(14)
*Z*	4
*D*_c_ (g·cm^−3^)	1.684
Absorption coefficient (mm^−1^)	1.377
*Θ* range for data collection (°)	3.281 to 26.021
*F* (000)	2016
*h*/*k*/*l* (min, max)	−19,19/−14,14/−26,26
Crystal size (mm)	0.15 × 0.21 × 0.24
Reflections collected	16726/7694 [*R*_int_ = 0.035]
Independent reflection	7694
*S*	1.033
Data/restraints/parameters	5555/54/581
Final *R* indices [*I* > 2*σ*(*I*)] ^a^ *R* indices (all data) ^b^	*R*_1_ = 0.0478 *wR*_2_ = 0.0738

**Table 3:** Selected bond distances (Å) and angles (°) for the Cu(II)–Na(I) complex.

**Bonds Lengths (Å)**			**Bonds Lengths (Å)**		
Cu1-O2	1.907(2)	Cu1-O5	1.903(2)	Cu1-O12	2.462(3)
Cu1-N1	2.002(3)	Cu1-N2	1.949(3)	Cu2-O6	1.910(2)
Cu2-O9	1.907(3)	Cu2-N3	1.945(3)	Cu2-N4	1.980(3)
Na1-O1	2.547(3)	Na1-O2	2.389(3)	Na1-O5	2.356(3)
Na1-O6	2.453(3)	Na1-O9	2.428(3)	Na1-O10	2.475(3)
Na1-O11	2.492(4)				
**Angle (°)**			**Angle (°)**		
O1-Na1-O2	62.93(9)	O1-Na1-O5	114.77(9)	O1-Na1-O6	125.89(10)
O1-Na1-O9	100.91(9)	O1-Na1-O11	127.1(12)	O2-Na1-O5	65.17(9)
O2-Na1-O6	125.67(9)	O2-Na1-O9	163.84(9)	O2-Na1-O10	109.08(9)
O5-Na1-O6	64.19(9)	O5-Na1-O9	126.70(9)	O5-Na1-O10	158.77(12)
O6-Na1-O9	62.91(9)	O6-Na1-O10	125.25(9)	O6-Na1-O11	106.71(9)
O9-Na1-O10	64.01(10)	O9-Na1-O11	108.71(9)	O10-Na1-O11	78.44(12)

**Table 4:** Hydrogen bondings (Å, deg) and C-H⋯π stacking interactions of Cu(II)–Na(I) complex.

D–H⋯A	d(D–H)	d(H⋯A)	d(D⋯A)	∠DHA
C8-H8A⋯O12	0.97	2.29	3.252(6)	171
O14-H14A⋯O12 ^#1^	0.82	2.37	3.075(6)	144
O14-H14A⋯O13 ^#1^	0.82	2.50	3.267(8)	157
C23-H23B⋯O13 ^#1^	0.97	2.31	2.987(9)	126
C15-H15⋯Cg1 ^#2^	0.96	2.79	3.473(5)	131

Symmetry codes: ^#1^ *x*, 1 + *y*, *z*; ^#2^ 1 − *x*, 1 − *y*, −*z*; Cg1 for the Cu(II)–Na(I) complex is the centroid of C1-C6 atoms.

## Supplementary Materials

Supplementary materials are available online.
