# Supplementary material for: Synthesis and Fluorescence Properties of Structurally Characterized Heterobimetalic Cu(II)–Na(I) Bis(salamo)-Based Complex Bearing Square Planar, Square Pyramid and Triangular Prism Geometries of Metal Centers
Source: Molecules. 2018 Apr 25;23(5):1006. doi: 10.3390/molecules23051006 (PMC6102540; doi:10.3390/molecules23051006)

checkCIF/PLATON report

You have not supplied any structure factors. As a result the full set of tests cannot be run.

THIS REPORT IS FOR GUIDANCE ONLY. IF USED AS PART OF A REVIEW PROCEDURE FOR PUBLICATION, IT SHOULD NOT REPLACE THE EXPERTISE OF AN EXPERIENCED CRYSTALLOGRAPHIC REFEREE.

No syntax errors found. CIF dictionary Interpreting this report

Datablock: d845

```
Bond precision:  C-C = 0.0055 A          Wavelength=0.71073

Cell:            a=15.8326(3)     b=11.4630(2)       c=21.7834(4)
                 alpha=90         beta=98.135(2)     gamma=90
Temperature:     155 K
```

	Calculated	Reported
Volume	3913.67(13)	3913.68(14)
Space group	P 21/n	P 21/n
Hall group	-P 2yn	-P 2yn
Moiety formula	C32 H28 Cu2 N5 Na O13, C H Cl3, C H4 O	C32 H28 Cu2 N5 Na O13, C H4 O, C H Cl3
Sum formula	C34 H33 Cl3 Cu2 N5 Na O14	C34 H33 Cl3 Cu2 N5 Na O14
Mr	992.09	992.07
Dx,g cm-3	1.684	1.684
Z	4	4
Mu (mm-1)	1.377	1.377
F000	2016.0	2016.0
F000’	2021.30	
h,k,lmax	19,14,26	19,14,26
Nref	7709	7694
Tmin,Tmax	0.726,0.813	0.845,1.000
Tmin’	0.711	

```
Correction method= # Reported T Limits: Tmin=0.845 Tmax=1.000
AbsCorr = MULTI-SCAN

Data completeness= 0.998          Theta(max)= 26.021

R(reflections)= 0.0478( 5555)     wR2(reflections)= 0.1112( 7694)

S = 1.027                  Npar= 581
```

The following ALERTS were generated. Each ALERT has the format
test-name_ALERT_alert-type_alert-level.
Click on the hyperlinks for more details of the test.

Alert level C

```
PLAT220_ALERT_2_C Non-Solvent  Resd 1  O   Ueq(max)/Ueq(min) Range       3.6 Ratio
PLAT230_ALERT_2_C Hirshfeld Test Diff for   O11      --N5        .       5.7 s.u.
PLAT234_ALERT_4_C Large Hirshfeld Difference O8       --C24             0.18 Ang.
PLAT241_ALERT_2_C High   'MainMol' Ueq as Compared to Neighbors of       O11 Check
PLAT241_ALERT_2_C High   'MainMol' Ueq as Compared to Neighbors of       O12 Check
PLAT242_ALERT_2_C Low    'MainMol' Ueq as Compared to Neighbors of        N5 Check
PLAT244_ALERT_4_C Low    'Solvent' Ueq as Compared to Neighbors of       C34 Check
PLAT410_ALERT_2_C Short Intra H...H Contact  H17      ..H22             1.98 Ang.
```

Alert level G

```
PLAT003_ALERT_2_G Number of Uiso or Uij Restrained non-H Atoms ...         9 Report
PLAT007_ALERT_5_G Number of Unrefined Donor-H Atoms .............          1 Report
PLAT012_ALERT_1_G No       _shelx_res_checksum Found in CIF ......    Please Check
PLAT042_ALERT_1_G Calc. and Reported MoietyFormula Strings  Differ    Please Check
PLAT186_ALERT_4_G The CIF-Embedded .res File Contains ISOR Records         2 Report
PLAT232_ALERT_2_G Hirshfeld Test Diff (M-X) Cu1      --O11     .         5.5 s.u.
PLAT300_ALERT_4_G Atom Site Occupancy of O7'        Constrained at       0.6 Check
PLAT300_ALERT_4_G Atom Site Occupancy of O8         Constrained at       0.6 Check
PLAT300_ALERT_4_G Atom Site Occupancy of O13        Constrained at      0.62 Check
PLAT300_ALERT_4_G Atom Site Occupancy of O7         Constrained at       0.4 Check
PLAT300_ALERT_4_G Atom Site Occupancy of O8'        Constrained at       0.4 Check
PLAT300_ALERT_4_G Atom Site Occupancy of O13'       Constrained at      0.38 Check
PLAT300_ALERT_4_G Atom Site Occupancy of C23        Constrained at       0.6 Check
PLAT300_ALERT_4_G Atom Site Occupancy of C24        Constrained at       0.6 Check
PLAT300_ALERT_4_G Atom Site Occupancy of C23'       Constrained at       0.4 Check
PLAT300_ALERT_4_G Atom Site Occupancy of C24'       Constrained at       0.4 Check
PLAT300_ALERT_4_G Atom Site Occupancy of H23A       Constrained at       0.6 Check
PLAT300_ALERT_4_G Atom Site Occupancy of H23B       Constrained at       0.6 Check
PLAT300_ALERT_4_G Atom Site Occupancy of H24A       Constrained at       0.6 Check
PLAT300_ALERT_4_G Atom Site Occupancy of H24B       Constrained at       0.6 Check
PLAT300_ALERT_4_G Atom Site Occupancy of H23C       Constrained at       0.4 Check
PLAT300_ALERT_4_G Atom Site Occupancy of H23D       Constrained at       0.4 Check
PLAT300_ALERT_4_G Atom Site Occupancy of H24C       Constrained at       0.4 Check
PLAT300_ALERT_4_G Atom Site Occupancy of H24D       Constrained at       0.4 Check
PLAT301_ALERT_3_G Main Residue  Disorder ..............(Resd  1  )        9% Note
PLAT333_ALERT_2_G Large Aver C6-Ring C-C Dist. C12      -C21            1.43 Ang.
PLAT395_ALERT_2_G Deviating  X-O-Y    Angle From 120 for O3            111.1 Degree
PLAT395_ALERT_2_G Deviating  X-O-Y    Angle From 120 for O8            112.7 Degree
PLAT395_ALERT_2_G Deviating  X-O-Y    Angle From 120 for O7            113.1 Degree
PLAT431_ALERT_2_G Short Inter HL..A Contact  Cl3      ..O13'            3.03 Ang.
PLAT860_ALERT_3_G Number of Least-Squares Restraints .............        54 Note
```

0 **ALERT level A** = Most likely a serious problem - resolve or explain
0 **ALERT level B** = A potentially serious problem, consider carefully
8 **ALERT level C** = Check. Ensure it is not caused by an omission or oversight
31 **ALERT level G** = General information/check it is not something unexpected

2 ALERT type 1 CIF construction/syntax error, inconsistent or missing data
13 ALERT type 2 Indicator that the structure model may be wrong or deficient
2 ALERT type 3 Indicator that the structure quality may be low
21 ALERT type 4 Improvement, methodology, query or suggestion
1 ALERT type 5 Informative message, check

It is advisable to attempt to resolve as many as possible of the alerts in all categories. Often the minor alerts point to easily fixed oversights, errors and omissions in your CIF or refinement strategy, so attention to these fine details can be worthwhile. In order to resolve some of the more serious problems it may be necessary to carry out additional measurements or structure refinements. However, the purpose of your study may justify the reported deviations and the more serious of these should normally be commented upon in the discussion or experimental section of a paper or in the "special_details" fields of the CIF. checkCIF was carefully designed to identify outliers and unusual parameters, but every test has its limitations and alerts that are not important in a particular case may appear. Conversely, the absence of alerts does not guarantee there are no aspects of the results needing attention. It is up to the individual to critically assess their own results and, if necessary, seek expert advice.

Publication of your CIF in IUCr journals

A basic structural check has been run on your CIF. These basic checks will be run on all CIFs submitted for publication in IUCr journals (*Acta Crystallographica*, *Journal of Applied Crystallography*, *Journal of Synchrotron Radiation*); however, if you intend to submit to *Acta Crystallographica Section C* or *E* or *IUCrData*, you should make sure that full publication checks are run on the final version of your CIF prior to submission.

Publication of your CIF in other journals

Please refer to the *Notes for Authors* of the relevant journal for any special instructions relating to CIF submission.

PLATON version of 30/01/2018; check.def file version of 30/01/2018

Datablock d845 - ellipsoid plot

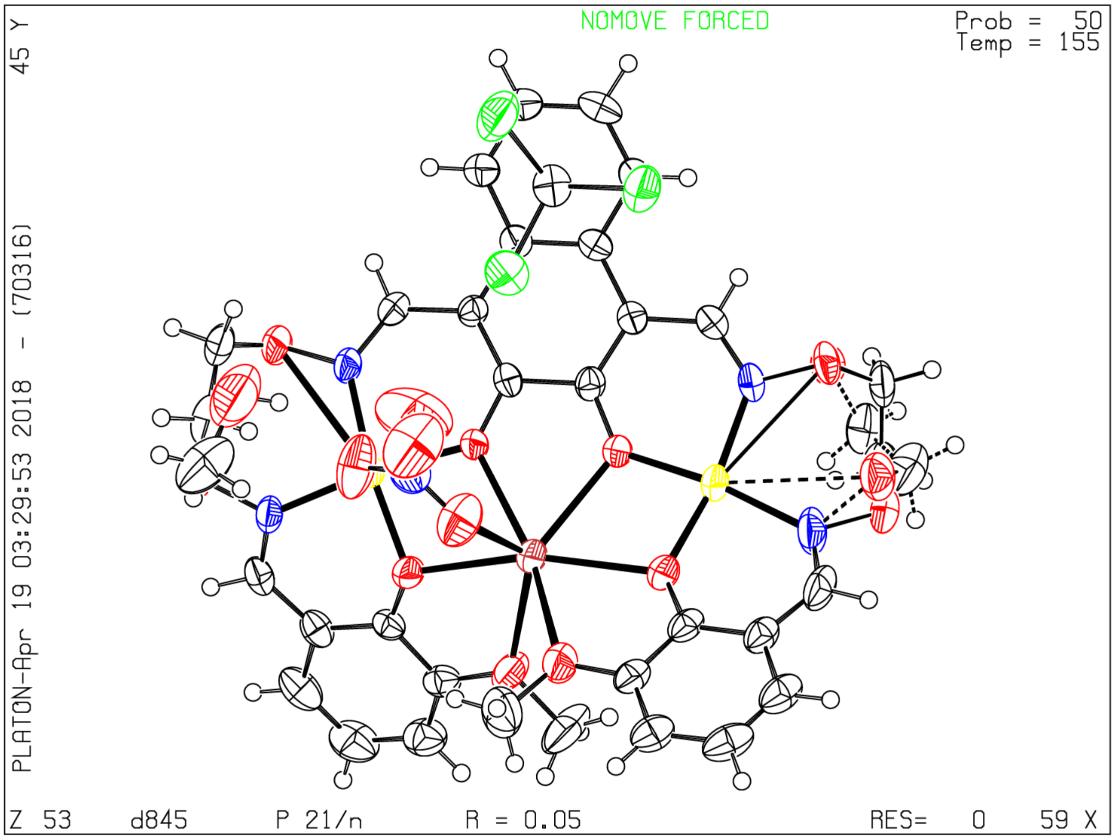

Supplement: Supplementary file 1 [file molecules-23-01006-s001.zip › Supplementary/Complex checkcif report.pdf]
